# Individual-Focused Interventions for Physician Burnout: A Meta-Analysis of Mindfulness, Coaching, and Peer Support

**DOI:** 10.3390/medicina62010039

**Published:** 2025-12-25

**Authors:** Akram Khan, Debbie Kim, Riannon Atwater, Raju Reddy

**Affiliations:** 1 Department of Medicine, Division of Pulmonary, Allergy and Critical Care Medicine, Oregon Health & Science University, Portland, OR 97239, USA; 2 Department of Surgery, Oregon Health & Science University, Portland, OR 97239, USA; kimde@ohsu.edu; 3 Department of Medicine, Oregon Health & Science University at Hillsboro Medical Center, Hillsboro, OR 97123, USA; atwaterr@ohsu.edu; 4 Department of Medicine, Division of Pulmonary and Critical Care Medicine, University of Texas at Austin, Austin, TX 78712, USA; raju.reddy@austin.utexas.edu

**Keywords:** physician burnout, Maslach burnout inventory, mindfulness, coaching, peer support, meta-analysis

## Abstract

*Background and Objectives*: Physician burnout, commonly described as emotional exhaustion (EE), depersonalization (DP), and reduced personal accomplishment (PA), remains common. We assessed whether structured, individual-focused programs improve Maslach Burnout Inventory (MBI) subscale scores among physicians. *Materials and Methods*: Registration, Open Science Framework, doi: 10.17605/OSF.IO/UAZ6B (unfunded). PubMed (MEDLINE) was searched from 1 January 2009 to 9 December 2023 (last searched 9 December 2023) to conduct a meta-analysis. Eligible English language studies evaluated a physician-focused intervention intended to reduce burnout and reported MBI outcomes; eligible designs were randomized trials, crossover trials, prospective cohort studies, or single-group pre–post studies. Risk of bias was rated using the original Cochrane Risk of Bias by two reviewers with consensus resolution. For quantitative synthesis, we pooled mean differences (MD) using fixed-effect inverse-variance meta-analysis with 95% confidence intervals (CI); heterogeneity was summarized with I^2^, and funnel plots were inspected qualitatively. *Results*: Of 2769 records, 17 studies met criteria for qualitative analysis, and 6 studies (*n* = 585 physicians; 273 intervention, 312 control) were pooled. Interventions included mindfulness curricula, professional coaching, or structured peer discussion groups. Compared with controls, interventions were associated with lower EE (MD −5.56; 95% CI, −6.68 to −4.44; I^2^ = 42%), lower DP (MD −2.11; 95% CI, −2.64 to −1.58), and higher PA (MD 2.01; 95% CI, 1.41 to 2.60). Funnel plots suggested asymmetry for EE. Evidence was limited by few trials, frequent high or unclear risk of bias in at least one domain, and variable intervention formats, and one pooled study used a single-group pre–post design. *Conclusions*: Structured individual-focused programs were associated with small but statistically significant changes in MBI subscale scores in physicians, but confidence in magnitude and generalizability are limited by study quality and a small evidence base. These programs may be useful adjuncts to organizational approaches to burnout.

## 1. Introduction

Physician burnout, commonly assessed with the Maslach Burnout Inventory (MBI), comprises 3 domains, emotional exhaustion, depersonalization and a diminished sense of personal accomplishment [1]. Emotional exhaustion is a loss of emotional energy, felt as fatigue and an inability to keep investing in patient care. Depersonalization is a detached or cynical attitude toward patients and colleagues. Reduced personal accomplishment is a sense of inefficacy and diminished competence [2]. Maslach and co-authors originally described burnout as a response to chronic occupational stress in helping professions, reflected in these three domains. In physicians, higher emotional exhaustion and depersonalization and lower personal accomplishment have been linked to harms for clinicians, patients, and health systems [3,4,5].

The COVID-19 pandemic coincided with higher reported burnout symptoms in many, but not all, physician groups. In a 2021 national survey, 62.8% of physicians reported at least one symptom of burnout, compared to 38.2% in 2020 [6]. Over the same period, satisfaction with work–life integration dropped from 46.1% to 30.2% [6]. However, the impact of the pandemic on burnout varied by specialty, practice setting, and gender. Burnout has been especially common among those working in high-acuity environments such as critical care and emergency medicine, and among women physicians (27% more likely to experience burnout) [1,6,7]. Beyond COVID-specific stressors, cross-sectional studies link burnout to structural factors including task load, documentation burden, staffing and scheduling pressures, and workplace climate [1,7,8,9,10]. United States physicians are ~82% more likely to experience burnout compared to other US workers, despite adjusting for demographics and work hours [2]. Burnout remains a significant threat to the healthcare workforce, contributing to an estimated $4.6 billion in annual economic losses due to physician turnover, reduced productivity, and compromised patient care [7,11,12].

Burnout in physicians is associated with depression, suicidal ideation, lower job satisfaction, early retirement, medical errors, poorer patient experience, and in some studies worse clinical outcomes [3,4,5]. At the same time, burnout is not a single, fully validated clinical diagnosis. Eckleberry-Hunt and colleagues have described how the MBI has been used with many cutoffs and ad hoc “burnout” thresholds, and how one-item questions such as “Are you burned out?” risk turning burnout into a vague label for general distress rather than a specific construct [12]. Rotenstein and colleagues reviewed more than 100 burnout studies and found extreme variability in definitions and prevalence, with over 140 distinct ways of defining “burnout” and wide ranges in reported rates [13]. These findings suggest that burnout, depression, and moral injury overlap but are not identical, and that current measures may not fully capture this complexity [12,13]. In this setting, MBI subscales are best viewed as continuous indicators of three related dimensions of emotional exhaustion, detachment or depersonalization, and perceived professional efficacy rather than as a binary diagnosis of “burned out” versus “not burned out.” Domain-level changes may reflect shifts in distress, disengagement, or moral strain to varying degrees, depending on the work environment and individual circumstances. Our analysis therefore focuses on continuous MBI scores, treating them as summary measures of these dimensions of physician distress while noting that they do not fully separate burnout from depression or moral injury.

Prior reviews suggest a role for both individual and system approaches, but the relative effects of specific strategies remain uncertain [11,14,15]. Individual programs (for example, mindfulness, coaching, and peer support) can improve coping and reduce emotional exhaustion, generally with modest effects, while system changes (for example, workload redesign, schedule flexibility, and supportive leadership) target structural drivers [16,17,18]. For many institutions, individual programs are among the most readily deployed options, even though they cannot substitute for system reform. In this manuscript, we treat individual-focused interventions as one part of a wider response to structurally driven burnout, not as stand-alone remedies. They are best viewed as ways to provide protected time, peer connection, and coaching while organizations work on workload, autonomy, electronic health record demands, staffing, and local culture.

Several systematic reviews have evaluated interventions for physician burnout. West and colleagues reported results from individual and organization-level programs for physicians using both randomized and pre–post study designs [14]. Panagioti et al. evaluated controlled interventions and contrasted physician-directed versus organization-directed approaches [11]. More recently, Haslam et al. limited inclusion to randomized trials involving physicians, physicians in training, and other health professionals and used random-effects models to pool changes in MBI domains [19]. Building on this literature, we focus specifically on physicians and on structured individual-level programs, mindfulness-based curricula, one-to-one or group coaching, and peer-support groups that report continuous MBI subscale scores. Our objective is to quantify domain-level changes in emotional exhaustion, depersonalization, and personal accomplishment associated with these commonly implemented interventions and to interpret those effects in light of the conceptual and measurement considerations outlined above.

This review does not evaluate organizational redesign interventions; rather, it estimates the expected magnitude of change in MBI domains associated with commonly deployed individual programs, recognizing that structural reforms are necessary for durable mitigation. Given ongoing institutional decisions about investing in coaching, mindfulness training, and peer-support models alongside system-level changes, an updated synthesis of these targeted interventions remains timely and directly relevant to practice.

## 2. Materials and Methods

### 2.1. Study Design and Study Selection

**Figure 1:**
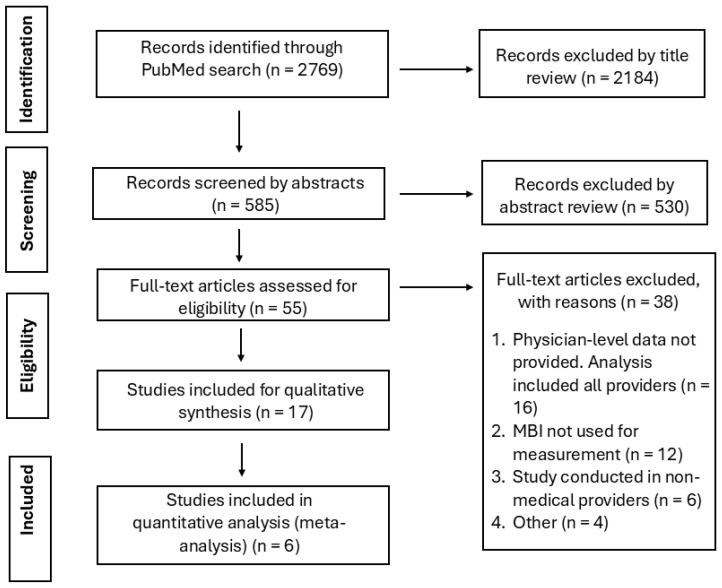
PRISMA flow diagram of study selection. The PubMed search identified 2769 records. After title screening, 2184 records were excluded as not meeting inclusion criteria, leaving 585 abstracts for review. Of these, 530 abstracts were excluded. 55 full-text articles were assessed for eligibility; 38 were excluded for predefined reasons (no physician-specific data, absence of MBI outcomes, mixed clinician samples without physician-only results, non-English language, or non-comparable design). Seventeen studies were included in the qualitative synthesis, and 6 trials contributed data to the quantitative meta-analysis.

This meta-analysis followed the Preferred Reporting Items for Systematic Reviews and Meta-Analyses (PRISMA) reporting guideline and the methodological framework outlined in the Cochrane Handbook of Systematic Reviews and Meta-Analysis [16,17,20]. This systematic review was registered on the Open Science Framework (OSF) (DOI: 10.17605/OSF.IO/UAZ6B; https://osf.io/mtq3u/). We searched PubMed (MEDLINE) from 1 January 2009 to 9 December 2023 (last searched 9 December 2023), using a structured strategy that combined MeSH terms for physicians and advanced practice clinicians (“Physicians,” “Physician Assistants,” and “Nurse Practitioners”), acute-care settings (“Critical Care,” “Intensive Care Units,” and “Emergency Service, Hospital”), and burnout-related constructs (“Burnout, Psychological,” “Burnout, Professional,” and “Resilience, Psychological”). The final PubMed search was executed on 9 December 2023. The complete PubMed search string, Boolean operators, and limits (humans, English language, article type, and date range), together with the PubMed search URL, are provided in the Online Supplement (Figure S1). We restricted the electronic search to PubMed because nearly all physician burnout intervention trials included in prior systematic reviews were indexed in this database, and we supplemented this search by screening reference lists of eligible studies and related systematic reviews to identify additional reports. Titles and abstracts were screened in 2 stages (title/abstract review followed by full-text assessment). The PubMed query reported in the Online Supplement (Figure S1) is the exact search used to generate the record counts shown in the PRISMA flow diagram (Figure 1), and all screening and study-selection steps were performed using those retrieved records. Three reviewers (DK, RA, and RR) independently screened records and evaluated full-text articles for eligibility; disagreements were resolved by discussion and consensus.

### 2.2. Inclusion and Exclusion Criteria

Studies were eligible for qualitative synthesis if they evaluated an intervention intended to reduce physician burnout and reported outcomes using the Maslach Burnout Inventory (MBI) [2]. The MBI assesses burnout across 3 subscales: emotional exhaustion (EE), depersonalization (DP), and personal accomplishment (PA) [2]. Eligible designs for the systematic review included randomized controlled trials (RCTs), crossover trials, prospective cohort studies, and single-group pre–post studies. For the quantitative meta-analysis, we pooled studies with extractable domain-level means and standard deviations at baseline and follow-up in physicians: RCTs and crossover trials plus 1 single-group pre–post study. Studies were excluded from the systematic review if they were not published in English or did not test a physician-focused intervention with MBI outcomes. Studies were excluded from quantitative pooling if they used non-standard MBI formats (e.g., the Dutch MBI or dichotomous-response formats) or did not report domain-level means and standard deviations.

### 2.3. Outcome Definition

The primary outcomes were changes in EE, DP, and PA scores on the MBI. We defined benefit as a reduction in EE or DP scores or an increase in PA scores. This approach reflects the range of burnout presentations and recognizes that improvements in any 1 domain may indicate clinical change [2]. 

### 2.4. Data Extraction

Three reviewers (DK, RA, and RR) independently extracted study-level data. Extracted variables included study design, setting, participant characteristics, intervention type and duration, and pre/post means and standard deviations for EE, DP, and PA. Where dispersion measures (standard deviation or standard error) were not reported or could not be derived from the published data, we did not impute values; such studies were retained for qualitative synthesis but were not included in quantitative pooling.

### 2.5. Risk of Bias Assessment

We assessed risk of bias for all intervention studies (randomized, nonrandomized, and single-group pre–post) using the original Cochrane Risk of Bias tool as a structured framework [21]. Domains assessed were random sequence generation and allocation concealment (selection bias), blinding of participants and personnel (performance bias), blinding of outcome assessment (detection bias), incomplete outcome data (attrition bias), selective outcome reporting (reporting bias), and other potential sources of bias. All assessments were conducted independently by 2 reviewers and reconciled by group consensus. For nonrandomized and single-group designs, items related to random sequence generation and allocation concealment were judged as high or unclear risk because no true random allocation occurred. Two reviewers independently rated each domain, and disagreements were resolved by consensus.

### 2.6. Statistical Analysis

We calculated pooled weighted mean differences (MDs) and 95% confidence intervals for each MBI subscale using the inverse variance methods. All outcomes were analyzed on the original MBI subscale scoring ranges; no scale transformations or score standardizations were performed prior to pooling. A 2-sided *p*-value < 0.05 was considered statistically significant. Between-study heterogeneity was assessed using the Cochrane Q test and the I^2^ statistic [22,23]. We interpreted I^2^ values < 30% as low, 30–50% as moderate, and > 50% as substantial heterogeneity [22]. Given the small number of studies and use of a common outcome scale (MBI), pooled MDs were estimated with a fixed-effect inverse variance model to summarize average differences across the included studies. We did not prespecify or conduct sensitivity analyses. We did not prespecify or conduct formal subgroup analyses or meta-regression because only six trials contributed to the pooled estimates. We did not formally rate certainty of evidence (e.g., using GRADE). Potential publication bias was examined by visual inspection of funnel plots; because fewer than 10 studies were available for each outcome, we did not perform formal tests for funnel plot asymmetry (such as Egger’s test) or trim-and-fill procedures and interpreted funnel plots qualitatively [24]. Analysis was performed analyses using Review Manager (RevMan), version 5.3 (The Cochrane Collaboration, London, UK, https://revman.cochrane.org) and Comprehensive Meta-Analysis v3 software (Biostat, Englewood, NJ, USA, https://www.meta-analysis.com).

## 3. Results

### 3.1. Study Selection

The PubMed search identified 2769 unique records (Figure 1). After title screening, 2184 records were excluded as unrelated to physician burnout interventions, leaving 585 abstracts for review. Of these, 530 abstracts were excluded because they did not meet inclusion criteria. We assessed 55 full-text articles for eligibility; 38 were excluded for the following reasons: no physician-specific data (*n* = 16), absence of MBI outcomes (*n* = 12), mixed clinician samples without physician-only results (*n* = 6), and other reasons, including lack of a control group, multiple intervention arms without separable data, or non-English language (*n* = 4).

Seventeen studies met criteria for inclusion in the final review (15 randomized controlled trials, one prospective cohort, and one pre-post study). Six studies provided complete MBI subscale mean and standard deviation data and were included in the quantitative meta-analysis: Krasner et al. (2009), Congiusta et al. (2020), Fainstad et al. (2022), West et al. (2021), Purdie et al. (2023) and Dyrbye et al. (2023) (Table 1) [25,26,27,28,29,30]. These trials enrolled 585 physicians, 273 in the intervention arm and 312 in the control arm.

The remaining 11 studies were not pooled because they used non-standard MBI formats, did not report domain-level means and standard deviations, or reported only change scores or prevalence; details and reasons for exclusion from the meta-analysis are summarized in Table S2.

### 3.2. Intervention Characteristics and Meta-Analysis Results

Krasner et al. conducted a single-group pre–post study involving 70 primary care physicians participating in a continuing medical education program focused on mindfulness, communication and self-awareness [25]. The intervention consisted of an 8-week mindfulness-based (2.5 h weekly and a 7-h retreat), followed by monthly maintenance sessions over 10 months [25]. This program included mindfulness meditation, self-awareness exercises, and narrative reflection. Emotional exhaustion decreased by 2.60 points (95% CI, −3.24 to −1.96), depersonalization by 6.77 points (95% CI, −8.17 to −5.37), and personal accomplishment increased by 2.4 points (95% CI, 1.7 to 3.10). Improvements in mindfulness were associated with lower emotional exhaustion and improved mood and empathy. Although the absence of a control group limits causal inference, the findings are consistent with benefit from sustained mindfulness training in this group of primary care physicians [25].

Congiusta et al. conducted a randomized controlled trial evaluating a 24-week online training program, The Clinician Experience Project, aimed at improving patient experience and reducing physician burnout [26]. 63 physicians from four specialties were randomized to intervention (*n* = 30) and control (*n* = 33) groups. The intervention included structured online modules and biweekly peer-led discussions. Participants in the intervention group reported improved patient experience scores (+1.40 vs. −0.11; *p* = 0.039) and reductions in depersonalization (MD = −3.60; *p* = 0.13) with increased personal accomplishment (MD = +1.50; *p* = 0.24). Nearly three-quarters (73.5%) of participants reported that the program improved their connections with colleagues and patients.

Fainstad et al. conducted a randomized clinical trial among 101 female resident physicians to assess the effectiveness of a six-month online group-coaching intervention, the Better Together program, in reducing burnout and improving well-being measures [27]. Fifty participants were assigned to the intervention and 51 to the control group. The primary outcome was burnout, assessed using the MBI, and secondary outcomes included impostor syndrome, self-compassion and moral injury. After 6 months, emotional exhaustion was lower in the intervention group (MD = −1.11; *p* = 0.17), although this difference was not statistically significant. No significant differences were found in depersonalization, personal accomplishment, or moral injury scores between groups. The program was associated with improved self-compassion and reduced impostor syndrome symptoms.

West et al. (2021) conducted a randomized controlled trial involving 125 practicing physicians at the Mayo Clinic to evaluate the *COMPASS* program, a six-month self-facilitated small-group peer-discussion intervention designed to support physician well-being [28]. Results showed significant reductions in emotional exhaustion (MD = −1.10) and depersonalization (MD = −2.10), with moderate improvement in personal accomplishment (MD = +1.20) [28].

Dyrbye et al. (2023) conducted a randomized clinical trial of 80 surgeons across Mayo Clinic sites to evaluate the effects of six individualized monthly professional coaching sessions on burnout, resilience, and quality of life [29]. Findings included reductions in emotional exhaustion (MD = −1.30) and depersonalization (MD = −4.10) [29].

Purdie et al. (2023) performed a randomized controlled trial of 66 resident physicians in the pediatric and medicine-pediatric residency programs at the UCLA Mattel Children’s Hospital [30]. They evaluated the effectiveness of hybrid (in-person and online) Mindful Awareness Practices (MAPs) curriculum on reducing stress in pediatric residents. Burnout was a secondary outcome and measured using MBI. Emotional exhaustion was reduced by 0.48 (−0.66–1.62), depersonalization was reduced by 0.37 (−0.77–1.51) and personal accomplishment changed by −0.25 (−1.17–0.67) [30].

The duration of interventions across the included studies varied, ranging from 6 weeks to 12 months with follow-up extending up to 18 months in some trials. All interventions aimed to reduce burnout and improve well-being, the delivery formats included web-based coaching, peer discussion groups (facilitated or self-facilitated) and individual professional coaching sessions. Despite differences in structure and intensity, most studies reported significant improvements in at least one domain of burnout, typically emotional exhaustion, with several also showing improvements in depersonalization, personal accomplishment, or related outcomes such as impostor syndrome and resilience.

**Figure 2:**
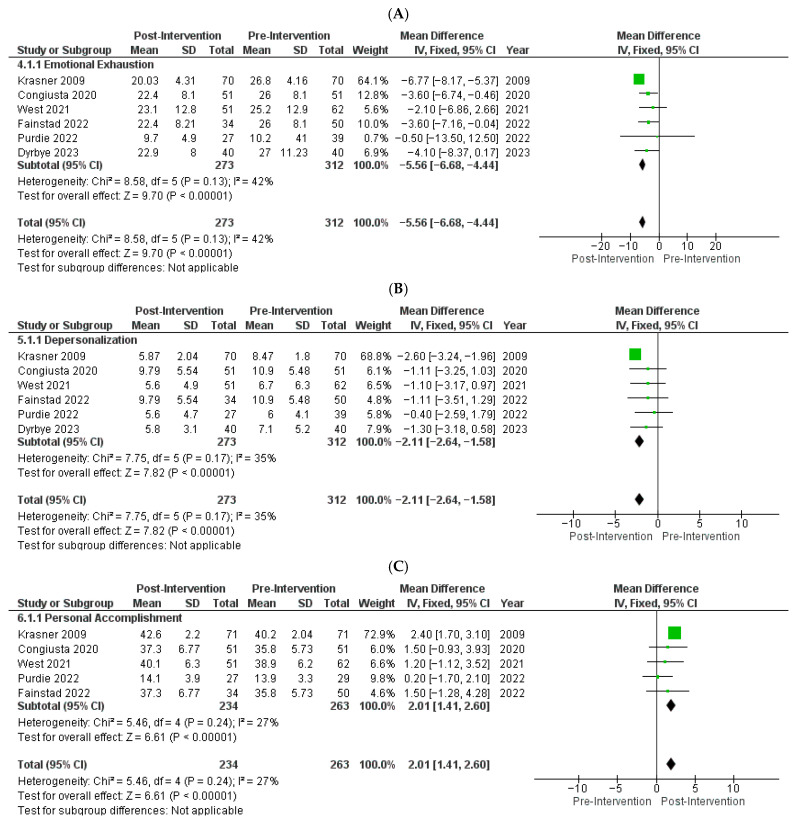
Forest plots of pooled effects of individual-focused interventions on Maslach Burnout Inventory (MBI) subscale scores using a fixed-effects inverse-variance model [25,26,27,28,29,30]. Each panel displays study-specific mean differences (MDs) with 95% confidence intervals (CIs); the pooled estimate is shown as a diamond: (**A**) Emotional Exhaustion (EE): Six studies (*n* = 585) showed lower EE with intervention vs. control (pooled MD, −5.56; 95% CI, −6.68 to −4.44; *p* < 0.00001), with moderate heterogeneity (I^2^ = 42%). (**B**) Depersonalization (DP): Six studies (*n* = 585) showed lower DP (pooled MD, −2.11; 95% CI, −2.64 to −1.58; *p* < 0.00001), with moderate heterogeneity (I^2^ = 35%). (**C**). Personal Accomplishment (PA): Five studies (*n* = 497) showed higher PA (pooled MD, 2.01; 95% CI, 1.41 to 2.60; *p* < 0.00001), with low heterogeneity (I^2^ = 27%).

Using a fixed-effect model, meta-analysis showed statistically significant changes in all three MBI domains, with reductions in EE and DP and improvement in PA. EE showed a pooled mean difference (MD) of −5.56 (95% CI: −6.68 to −4.44; *p* < 0.00001; Figure 2A). DP was reduced by −2.11 points (95% CI: −2.64 to −1.58; *p* < 0.00001; Figure 2B). PA improved with a pooled MD of +2.01 (95% CI: 1.41 to 2.60; *p* < 0.00001; Figure 2C). With few pooled studies and differences in intervention structure, these pooled estimates should be read as average differences across the included studies rather than definitive effect sizes. No sensitivity analyses were performed.

Funnel plots showed moderate asymmetry for emotional exhaustion and near-symmetric patterns for depersonalization and personal accomplishment (Figure S2). Across the 17 included studies, most trials were judged to have high or unclear risk of bias in at least 1 domain related to randomization or blinding, whereas attrition and selective reporting were usually judged to be low risk (Figure S3, Table S1). A study-level summary of risk-of-bias judgments for each domain is provided in Table S1. We then reviewed conceptually relevant studies that were not eligible for pooling; Table S2 lists each study with the specific reason for exclusion from the quantitative analysis and certainty of evidence was not formally rated.

### 3.3. Excluded Studies’ Characteristics

Weight et al. tested an elective, team-based, incentivized exercise program for physicians and staff [31]. They assessed physical activity, quality of life, and burnout at baseline and 3 months. Physical activity was compared with the Department of Health and Human Services recommendations for exercise, quality of life, and burnout was measured using a modified MBI using EE and DP subscales. There was no statistically significant difference in burnout between participants and non-participants. Because the study used a modified MBI format and did not report domain-level means and standard deviations, it could not be included in the pooled analysis.

Milstein et al. assessed burnout in house officers and examined whether individual psychotherapy could reduce symptoms [32]. The investigators reported no statistically significant differences in burnout outcomes between the control and intervention groups, but no raw MBI data were presented, precluding inclusion in the quantitative synthesis.

Martins et al. studied 74 pediatric residents who participated in self-care workshops for 2 months, using pre- and post- MBI scores [33]. MBI scores were grouped into low, medium and high symptoms. Individuals who scored medium to high in 2 out of 3 categories were defined as experiencing burnout. Their intervention did not reduce burnout although they reported a slight improvement in depersonalization. They did not report raw MBI data, preventing this study from being included in the meta-analysis.

Amutio et al. conducted a 2-phase mindfulness-based stress reduction program for 42 hospital physicians, consisting of an initial 8-week course followed by a 10-month maintenance phase [34]. Outcomes included the Five Facets of Mindfulness Questionnaire, the MBI, and cardiovascular measures (heart rate and blood pressure). After 8 weeks, the intervention group showed significant reductions in emotional exhaustion and improvements in mindfulness and cardiovascular parameters, with effect sizes increasing over the maintenance phase. Because the study used a Spanish adaptation of the MBI rather than a standard MBI format with domain-level scores, it was not eligible for inclusion in the meta-analysis.

Ripp et al. performed a randomized clinical trial of a facilitated discussion group intervention for 51 first-year internal medicine residents, with twice-monthly sessions addressing stress, work–life balance, and job satisfaction [35]. Burnout was assessed using a modified MBI looking at EE and DP scores but did not report individual scores, rather attributing high scores to a positive burnout. Baseline burnout prevalence and end-of-year incidence were similar in the intervention and control groups, and the intervention did not reduce burnout or improve secondary outcomes. Because MBI results were reported only as categorical burnout prevalence without domain-level means and standard deviations, this study could not be included in the pooled analysis.

Similarly, Verweij et al. conducted a randomized clinical trial of Mindfulness-Based Stress Reduction on burnout in residents using the Dutch version of the MBI [36]. They found no significant difference in emotional exhaustion but reported improvement in personal accomplishment, mindfulness skills, self-compassion, and empathy. Because this trial used the Dutch MBI rather than an English-language MBI version, it was excluded from the quantitative synthesis.

Medisauskaite et al. conducted a randomized controlled trial of a brief psychoeducational intervention for 227 physicians that delivered online modules on burnout, stress, coping with patient death, and management of work-related distress, with outcomes assessed 7 days after exposure [37]. The intervention reduced emotional exhaustion, depersonalization, and anxiety compared with control conditions, with no short-term change in other health or behavior outcomes. MBI results were reported as aggregated change scores across several intervention arms without arm-specific means or standard deviations, so this trial could not be incorporated into the quantitative meta-analysis.

A separate study examined the effects of virtual reality on stress reduction and burnout in otolaryngology residents. Weitzman et al. conducted a prospective randomized crossover trial in which residents used weekly virtual reality-guided mindfulness meditation and paced breathing during a clinical rotation and observed a decrease in emotional exhaustion on the MBI compared with periods without virtual reality use [38]. Because the article reported only change scores and *p* values, without group-specific means and standard deviations, it was not eligible for inclusion in our meta-analysis.

McGonagle et al. tested a psychology-based coaching program for primary care physicians in a randomized trial [39]. They found that coaching reduced burnout and improved well-being, but burnout was analyzed as a composite index, and domain-specific emotional exhaustion, depersonalization, and personal accomplishment results were not reported, precluding extraction of subscale values for this review.

Another RCT by Hata et al. evaluated the results of self-facilitated groups for physicians, nurse practitioners, and nurse midwives [40]. Emotional exhaustion and depersonalization declined over the three-month program, and overall burnout and work engagement improved in the combined sample. However, burnout outcomes were reported using single-item measures and a continuous composite score across all professions, without separate MBI subscale means for physicians, so this trial was not included in the pooled analysis.

In a study looking at moral case deliberation (structured moral dialogue around ethical difficulties in practice) Kok et al. enrolled multidisciplinary ICU teams in a parallel cluster randomized trial with 21-month follow-up [41]. structured moral case deliberation had little effect on emotional exhaustion or depersonalization and was associated with a small decrease in personal accomplishment, while moral distress and team climate improved. Because outcomes were reported for all ICU professionals combined rather than for physicians alone, this trial was excluded from the quantitative synthesis.

## 4. Discussion

In this meta-analysis of structured, individual-focused programs for physicians, we observed small improvements in emotional exhaustion, depersonalization, and professional accomplishment as measured by MBI subscale scores. The magnitude of change was similar to effects reported in prior reviews of physicians and mixed clinician samples [11,14,19]. Although the pooled mean differences were modest on the MBI’s unweighted scales, West et al. noted that even 1-point changes in MBI domain scores have been associated with meaningful differences in adverse outcomes and that cut points separating “average” from “high” scores are narrow (e.g., DP 6–9 vs. ≥10), such that 1–2 point shifts may be meaningful for some individuals [14]. Consistent with this framing, our findings suggest that mindfulness-based curricula, coaching, and peer groups are associated with incremental improvements in distress and engagement rather than stand-alone remedies for structurally driven burnout. These programs alone are unlikely to fully reverse burnout without concurrent changes in workload, practice structure, and organizational culture. Peer connection was a recurring feature: interventions that included structured discussion (for example, small-group or peer-led sessions) were associated with reductions in emotional exhaustion and depersonalization, likely by strengthening interpersonal support, normalization of shared experience, and reduced isolation in high-stress environments [8]. Given conceptual overlap between burnout, depression, and moral injury, these findings are best interpreted as modest reductions in exhaustion and detachment and small gains in perceived professional efficacy, rather than as resolution of a single, uniform burnout diagnosis.

The available evidence is consistent with a combined approach. Individual-directed programs may support clinicians, but they are unlikely to address the structural drivers of burnout in isolation. Prior work has linked administrative streamlining, reduced documentation burden, improve EHR usability, and team-based care with lower emotional exhaustion and better job satisfaction [11,14,15,19]. Leadership support and alignment between physician values and organizational culture have also been associated with lower burnout [3,4]. Together these findings suggest that durable improvement likely depends on addressing both individual coping and organizational conditions; however, the modest effect sizes and limited number of trials in the present meta-analysis do not support strong inferences about the optimal mix, sequencing, or comparative effectiveness of organizational vs. individual strategies. Our pooled results should therefore be interpreted as the incremental benefit in trials of individual-focused programs, not as evidence that individual coping strategies can resolve system-level causes of distress.

Several gaps remain. It is unclear whether multimodal programs (those combining mindfulness, coaching, and peer support) offer additive benefit or unnecessary complexity. Further, differences by gender, specialty, and training level require additional study. Early-career physicians often face greater workload and lower autonomy, while women physicians may experience stronger effects from value mismatch [10,42]. Future trials designed to evaluate differential effects across subgroups and settings would strengthen the evidence base and improve implementation relevance.

A persistent barrier to synthesis is variability in burnout assessment and reporting. Although the MBI is widely used, trials differ in version, scoring methods, definitions of burnout severity, and reporting of domain level means and dispersion [2]. This heterogeneity limits comparability across trials and complicates the interpretation of pooled estimates [12,13]. We therefore pooled continuous MBI subscale scores rather than dichotomous burnout categories to reduce this problem by focusing on domain-level changes in exhaustion, depersonalization, and perceived accomplishment, while noting that these domains only partly capture the full spectrum of physician distress.

This study has limitations. Interventions, study populations, and follow-up varied, contributing to heterogeneity in observed effects. Many studies were small and may have been underpowered for domain-level outcomes. Loss to follow-up was common limiting inferences regarding durability. We used a fixed-effect model for the pooled analyses; given moderate heterogeneity (I^2^ ≈ 42% for emotional exhaustion) and only six trials, these pooled estimates likely understate between-study variability and should be interpreted cautiously. We therefore frame the observed MBI changes as small and hypothesis-generating rather than definitive estimates of treatment effect. In addition, one pooled study used a single-group pre–post design rather than randomization, which introduces design-related bias; we retained it to preserve information on domain-level changes but note that this choice may further limit causal inference. Finally, most studies were conducted in academic settings, which may limit applicability to community-based physicians or those practicing in non-academic environments. Non-English studies were excluded, which may limit generalizability and introduce selection bias.

Despite these limitations, this meta-analysis has several strengths. We focused on physician populations and structured individual-focused interventions, improving interpretability relative to broader reviews that combine multiple clinician types. We included studies across a range of specialties and career stages, including residents and attending physicians, and interventions ranging from in-person curricula to digital and coaching-based formats, supporting practical relevance across implementation contexts.

Future research should prioritize longitudinal studies of multimodal programs, develop targeted strategies based on gender and career stage, and adopt standardized burnout assessment tools to improve comparability across studies. Additional meta-analyses that include studies in non-English languages, could broaden the evidence base and improve relevance across health systems.

## 5. Conclusions

This meta-analysis found that individual-focused programs for physicians, mindfulness-based curricula, professional coaching, and peer discussion groups were associated with small but statistically significant improvements in MBI subscale scores for emotional exhaustion, depersonalization, and personal accomplishment. The magnitude of change was modest, and clinical importance remains uncertain, but the findings are consistent with prior synthesis of clinician burnout interventions. These results support interpreting individual programs as complementary supports that may yield incremental improvements in distress and engagement, rather than substitutes for organizational reforms targeting workload, documentation burden, autonomy, staffing and workplace culture.

Measurement remains a limiting factor. Trials used different MBI versions and scoring approaches, frequently reported incomplete domain-level data, and rarely provided sufficient information to support robust comparisons across programs. Future studies should report domain means and measures of spread at baseline and follow-up, specify the MBI version and language used, and describe handling of missing data and consider harmonized reporting standards to improve comparability.

## Figures and Tables

**Table 1:** Characteristics and interventions of included studies reporting Maslach Burnout Inventory (MBI) outcomes in the meta-analysis. Abbreviations: AMC, academic medical center; MBI, Maslach Burnout Inventory; RCT, randomized clinical trial; CME, continuing medical education; IM, internal medicine; Peds, pediatrics; Med–Peds, internal medicine–pediatrics; MAPs, Mindful Awareness Practices; Int, intervention; Ctrl, control; F, female; M, male; AZ, Arizona; CO, Colorado; FL, Florida; MN, Minnesota; NY, New York; WI, Wisconsin; USA, United States of America. Single-group pre–post denotes a within-participant design without a randomized control group.

Author, Year	Location	Study Design	Inclusion Criteria	Health Care Setting	Number of Females (F) Number of Males (M)	Age Range, Years	Intervention	Intervention Duration	Control	Follow Up
Krasner 2009 [25]	Rochester, NY, USA	Pre- and post-	Primary care physicians (family medicine, IM, peds, or combined med-peds with RIPA revenues > $20,000	Primary care practices	n 70 (32 F, 38 M)	Not Reported	8-week CME mindfulness course (2.5 h/week, plus 7-h retreat) followed by a 10-month maintenance (2.5 h/month); included mindfulness meditation, narratives, and discussion.	52 weeks	(within-participant comparison)	2, 12, and 15 months
Congiusta 2020 [26]	New York City, NY, USA	RCT	Physicians in IM, cardiology, obstetrics/gynecology & surgery with middle tertile patient experience scores.	Hospital Group	n 66 Int (n 30, 11 F/19 M) Ctrl (n 33, 13 F/20 M)	33–97 years	24-week online program with video modules, coaching tips, biweekly discussion calls, and social events to improve communication, burnout, and patient experience.	24 weeks	No intervention; usual patient care practices	24 weeks (post intervention)
Fainstad 2022 [27]	Aurora, CO, USA	RCT	Female resident physicians	AMC	n 101 all F (Int 50, Ctrl 51)	25–35 years	6-month online group coaching, including live coaching calls, written anonymous coaching, and self-study modules on goal setting, impostor syndrome, and feedback.	6 months	Usual residency training	6 months (post intervention)
West 2021 [28]	Rochester, MN, USA	RCT	IM Physicians	AMC	n 125 53 F/70 M (Int 64, Ctrl 61)	<30–>60 (categorical)	12 bi-weekly self-facilitated discussion groups on meaning in work and peer support.	6 months	Usual practice	6 months (post intervention)
Dyrbye 2023 [29]	Mayo Clinic (AZ, FL, MN, and WI).	RCT	Practicing surgeons in general surgery & surgical subspecialties.	AMC	n 80 Int: (n 40, 16 F/24 M; Ctrl: (n 40, 16 F/24 M)	31–61+ (categorical)	6 monthly one-to-one sessions with a professional coach (well-being and professional development)	5 months	Delayed coaching (usual practice during trial)	5 months (post intervention)
Purdie 2023 [30]	Los Angeles, California, USA	RCT	Resident physician in peds and med-peds	AMC	n 66 52 F/14 M (Int 27, Ctrl 39)	25–34 years	One in-person 60–min session and 6-week access to a digitally delivered MAPs curriculum or wait-list control.	6 weeks	Wait-list control	2 weeks after 6-week intervention

## Data Availability

The original contributions presented in this study are included in the article/Supplementary Material. Further inquiries can be directed to the corresponding author.

## Supplementary Materials

The following supporting information can be downloaded at https://www.mdpi.com/article/10.3390/medicina62010039/s1. Figure S1. PubMed search strategy (online supplement); Figure S2. Funnel plots assessing small-study effects for Maslach Burnout Inventory (MBI) subscales; Figure S3. Risk of bias (RoB) summary for intervention studies; Table S1. Risk of bias judgments for intervention studies; Table S2. Characteristics and interventions of studies excluded from meta-analysis due to methodological limitations.

## Abbreviations

The following abbreviations are used in this manuscript:

MBI	Maslach Burnout Inventory
EE	Emotional Exhaustion
DP	Depersonalization
PA	Personal Accomplishment
RCT	Randomized Controlled Trial
MD	Mean Difference
CI	Confidence Interval
PRISMA	Preferred Reporting Items for Systematic Reviews and Meta-Analyses
I^2^	I-squared Statistic (measure of heterogeneity)
US	United States
COVID-19	Coronavirus Disease 2019

## References

[1] Klick J.C., Syed M., Leong R., Miranda H., Cotter E.K. (2023). Health and Well-Being of Intensive Care Unit Physicians: How to Ensure the Longevity of a Critical Specialty. Anesthesiol. Clin..

[2] Maslach C., Jackson S., Leiter M. (1997). The Maslach Burnout Inventory Manual. Evaluating Stress: A Book of Resources.

[3] Gregory S.T., Menser T. (2015). Burnout Among Primary Care Physicians: A Test of the Areas of Worklife Model. J. Healthc. Manag..

[4] Shanafelt T.D., Noseworthy J.H. (2017). Executive Leadership and Physician Well-being: Nine Organizational Strategies to Promote Engagement and Reduce Burnout. Mayo Clin. Proc..

[5] Tawfik D.S., Profit J., Morgenthaler T.I., Satele D.V., Sinsky C.A., Dyrbye L.N., Tutty M.A., West C.P., Shanafelt T.D. (2018). Physician Burnout, Well-being, and Work Unit Safety Grades in Relationship to Reported Medical Errors. Mayo Clin. Proc..

[6] Shanafelt T.D., West C.P., Dyrbye L.N., Trockel M., Tutty M., Wang H., Carlasare L.E., Sinsky C. (2022). Changes in Burnout and Satisfaction with Work-Life Integration in Physicians During the First 2 Years of the COVID-19 Pandemic. Mayo Clin. Proc..

[7] Harry E., Sinsky C., Dyrbye L.N., Makowski M.S., Trockel M., Tutty M., Carlasare L.E., West C.P., Shanafelt T.D. (2021). Physician Task Load and the Risk of Burnout Among US Physicians in a National Survey. Jt. Comm. J. Qual. Patient Saf..

[8] Niven A.S., Sessler C.N. (2022). Supporting Professionals in Critical Care Medicine: Burnout, Resiliency, and System-Level Change. Clin. Chest Med..

[9] Olson K., Sinsky C., Rinne S.T., Long T., Vender R., Mukherjee S., Bennick M., Linzer M. (2019). Cross-sectional survey of workplace stressors associated with physician burnout measured by the Mini-Z and the Maslach Burnout Inventory. Stress. Health.

[10] Sterling R., Rinne S.T., Reddy A., Moldestad M., Kaboli P., Helfrich C.D., Henrikson N.B., Nelson K.M., Kaminetzky C., Wong E.S. (2022). Identifying and Prioritizing Workplace Climate Predictors of Burnout Among VHA Primary Care Physicians. J. Gen. Intern. Med..

[11] Panagioti M., Panagopoulou E., Bower P., Lewith G., Kontopantelis E., Chew-Graham C., Dawson S., van Marwijk H., Geraghty K., Esmail A. (2017). Controlled Interventions to Reduce Burnout in Physicians: A Systematic Review and Meta-analysis. JAMA Intern. Med..

[12] Eckleberry-Hunt J., Kirkpatrick H., Barbera T. (2018). The Problems with Burnout Research. Acad. Med..

[13] Rotenstein L.S., Torre M., Ramos M.A., Rosales R.C., Guille C., Sen S., Mata D.A. (2018). Prevalence of Burnout Among Physicians: A Systematic Review. Jama.

[14] West C.P., Dyrbye L.N., Erwin P.J., Shanafelt T.D. (2016). Interventions to prevent and reduce physician burnout: A systematic review and meta-analysis. Lancet.

[15] DeChant P.F., Acs A., Rhee K.B., Boulanger T.S., Snowdon J.L., Tutty M.A., Sinsky C.A., Thomas Craig K.J. (2019). Effect of Organization-Directed Workplace Interventions on Physician Burnout: A Systematic Review. Mayo Clin. Proc. Innov. Qual. Outcomes.

[16] Cumpston M., Li T., Page M.J., Chandler J., Welch V.A., Higgins J.P., Thomas J. (2019). Updated guidance for trusted systematic reviews: A new edition of the Cochrane Handbook for Systematic Reviews of Interventions. Cochrane Database Syst. Rev..

[17] Liberati A., Altman D.G., Tetzlaff J., Mulrow C., Gøtzsche P.C., Ioannidis J.P., Clarke M., Devereaux P.J., Kleijnen J., Moher D. (2009). The PRISMA statement for reporting systematic reviews and meta-analyses of studies that evaluate health care interventions: Explanation and elaboration. J. Clin. Epidemiol..

[18] Tarantino B., Earley M., Audia D., D’Adamo C., Berman B. (2013). Qualitative and quantitative evaluation of a pilot integrative coping and resiliency program for healthcare professionals. Explore.

[19] Haslam A., Tuia J., Miller S.L., Prasad V. (2024). Systematic Review and Meta-Analysis of Randomized Trials Testing Interventions to Reduce Physician Burnout. Am. J. Med..

[20] Page M.J., McKenzie J.E., Bossuyt P.M., Boutron I., Hoffmann T.C., Mulrow C.D., Shamseer L., Tetzlaff J.M., Akl E.A., Brennan S.E. (2021). The PRISMA 2020 statement: An updated guideline for reporting systematic reviews. BMJ.

[21] Higgins J.P., Altman D.G., Gøtzsche P.C., Jüni P., Moher D., Oxman A.D., Savovic J., Schulz K.F., Weeks L., Sterne J.A. (2011). The Cochrane Collaboration’s tool for assessing risk of bias in randomised trials. BMJ.

[22] Higgins J.P., Thompson S.G. (2002). Quantifying heterogeneity in a meta-analysis. Stat. Med..

[23] Higgins J.P., Thompson S.G., Deeks J.J., Altman D.G. (2003). Measuring inconsistency in meta-analyses. BMJ.

[24] Egger M., Davey Smith G., Schneider M., Minder C. (1997). Bias in meta-analysis detected by a simple, graphical test. BMJ.

[25] Krasner M.S., Epstein R.M., Beckman H., Suchman A.L., Chapman B., Mooney C.J., Quill T.E. (2009). Association of an Educational Program in Mindful Communication with Burnout, Empathy, and Attitudes Among Primary Care Physicians. JAMA.

[26] Congiusta S., Ascher E.M., Ahn S., Nash I.S. (2020). The Use of Online Physician Training Can Improve Patient Experience and Physician Burnout. Am. J. Med. Qual..

[27] Fainstad T., Mann A., Suresh K., Shah P., Dieujuste N., Thurmon K., Jones C.D. (2022). Effect of a Novel Online Group-Coaching Program to Reduce Burnout in Female Resident Physicians: A Randomized Clinical Trial. JAMA Netw. Open.

[28] West C.P., Dyrbye L.N., Satele D.V., Shanafelt T.D. (2021). Colleagues Meeting to Promote and Sustain Satisfaction (COMPASS) Groups for Physician Well-Being: A Randomized Clinical Trial. Mayo Clin. Proc..

[29] Dyrbye L.N., Gill P.R., Satele D.V., West C.P. (2023). Professional Coaching and Surgeon Well-being: A Randomized Controlled Trial. Ann. Surg..

[30] Purdie D.R., Federman M., Chin A., Winston D., Bursch B., Olmstead R., Bulut Y., Irwin M.R. (2023). Hybrid Delivery of Mindfulness Meditation and Perceived Stress in Pediatric Resident Physicians: A Randomized Clinical Trial of In-Person and Digital Mindfulness Meditation. J. Clin. Psychol. Med. Settings.

[31] Weight C.J., Sellon J.L., Lessard-Anderson C.R., Shanafelt T.D., Olsen K.D., Laskowski E.R. (2013). Physical activity, quality of life, and burnout among physician trainees: The effect of a team-based, incentivized exercise program. Mayo Clin. Proc..

[32] Milstein J.M., Raingruber B.J., Bennett S.H., Kon A.A., Winn C.A., Paterniti D.A. (2009). Burnout assessment in house officers: Evaluation of an intervention to reduce stress. Med. Teach..

[33] Martins A.E., Davenport M.C., Del Valle M.P., Di Lalla S., Domínguez P., Ormando L., Ingratta A., Gambarini H., Ferrero F. (2011). Impact of a brief intervention on the burnout levels of pediatric residents. J. Pediatr..

[34] Amutio A., Martínez-Taboada C., Delgado L.C., Hermosilla D., Mozaz M.J. (2015). Acceptability and Effectiveness of a Long-Term Educational Intervention to Reduce Physicians’ Stress-Related Conditions. J. Contin. Educ. Health Prof..

[35] Ripp J.A., Fallar R., Korenstein D. (2016). A Randomized Controlled Trial to Decrease Job Burnout in First-Year Internal Medicine Residents Using a Facilitated Discussion Group Intervention. J. Grad. Med. Educ..

[36] Verweij H., van Ravesteijn H., van Hooff M.L.M., Lagro-Janssen A.L.M., Speckens A.E.M. (2018). Mindfulness-Based Stress Reduction for Residents: A Randomized Controlled Trial. J. Gen. Intern. Med..

[37] Medisauskaite A., Kamau C. (2019). Reducing burnout and anxiety among doctors: Randomized controlled trial. Psychiatry Res..

[38] Weitzman R.E., Wong K., Worrall D.M., Park C., McKee S., Tufts R.E., Teng M.S., Iloreta A.M. (2021). Incorporating Virtual Reality to Improve Otolaryngology Resident Wellness: One Institution’s Experience. Laryngoscope.

[39] McGonagle A.K., Schwab L., Yahanda N., Duskey H., Gertz N., Prior L., Roy M., Kriegel G. (2020). Coaching for primary care physician well-being: A randomized trial and follow-up analysis. J. Occup. Health Psychol..

[40] Hata S.R., Berkowitz L.R., James K., Simpkin A.L. (2022). An Interprofessional Group Intervention to Promote Faculty Well-Being: A Randomized Clinical Trial. J. Contin. Educ. Health Prof..

[41] Kok N., Zegers M., Teerenstra S., Fuchs M., van der Hoeven J.G., van Gurp J.L.P., Hoedemaekers C.W.E. (2023). Effect of Structural Moral Case Deliberation on Burnout Symptoms, Moral Distress, and Team Climate in ICU Professionals: A Parallel Cluster Randomized Trial. Crit. Care Med..

[42] Leiter M.P., Frank E., Matheson T.J. (2009). Demands, values, and burnout: Relevance for physicians. Can. Fam. Physician.

