# Supplementary material for: Individual-Focused Interventions for Physician Burnout: A Meta-Analysis of Mindfulness, Coaching, and Peer Support
Source: Medicina (Kaunas). 2025 Dec 25;62(1):39. doi: 10.3390/medicina62010039 (PMC12843167; doi:10.3390/medicina62010039)
Supplement: Supplementary file 1 [file medicina-62-00039-s001.zip › medicina-3936358-supplementary.pdf]

Online Supplement Individual-Focused Interventions for Physician Burnout: A Meta-analysis of Mindfulness, Coaching, and Peer Support

Figure S1. PubMed search strategy (online supplement).

PubMed (MEDLINE) was searched on 09 December 2023 using the following MeSH-based strategy: ("Physicians"[MeSH] OR "Physician Assistants"[MeSH] OR "Nurse Practitioners"[MeSH]) AND ("Critical Care"[MeSH] OR "Intensive Care Units"[MeSH] OR "Emergency Service, Hospital"[MeSH]) AND ("Burnout, Psychological"[MeSH] OR "Burnout, Professional"[MeSH] OR "Resilience, Psychological"[MeSH]). Results were limited to Humans, English language, and publication dates from 1 January 2009 through 9 December 2023, and filtered by publication type (Clinical Study, Clinical Trial, Randomized Controlled Trial, Meta-Analysis, Review, and Systematic Review).

Source: https://bit.ly/4iYoeI7

Figure S2. Funnel plots assessing small-study effects for Maslach Burnout Inventory (MBI) subscales.

Funnel plots display mean difference (MD) on the x-axis versus the standard error of the MD (SE[MD]) on the y-axis for studies included in each MBI subscale meta-analysis. The solid vertical line indicates the pooled MD; diagonal lines indicate pseudo 95% confidence limits around the pooled estimate. Because each subscale analysis includes fewer than 10 studies, funnel-plot interpretation is descriptive and underpowered to distinguish chance asymmetry from small-study effects (e.g., publication bias or other reporting biases).

A. Emotional Exhaustion (EE).

B. Depersonalization (DP).

C. Personal Accomplishment (PA).

A. Emotional Exhaustion (EE).

A.

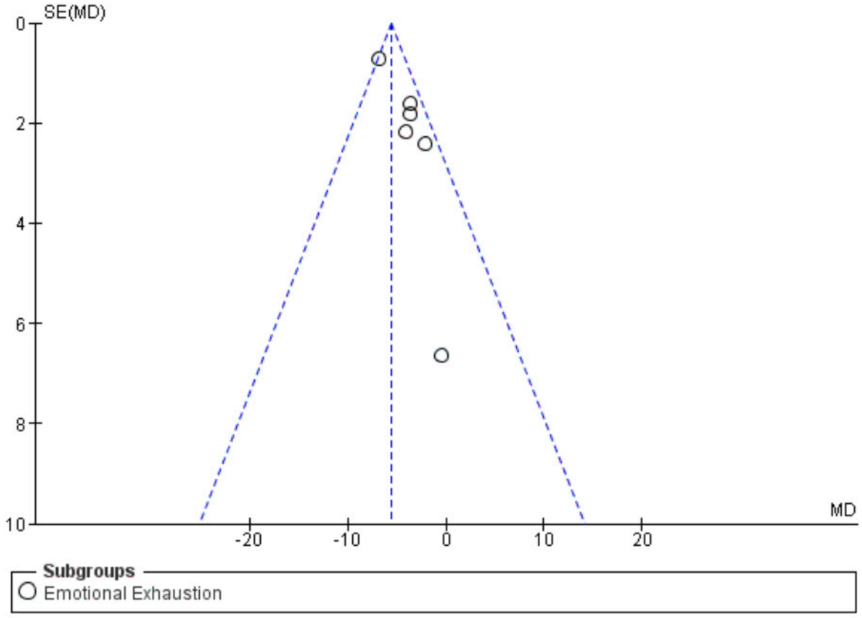

B. Depersonalization (DP).

B.

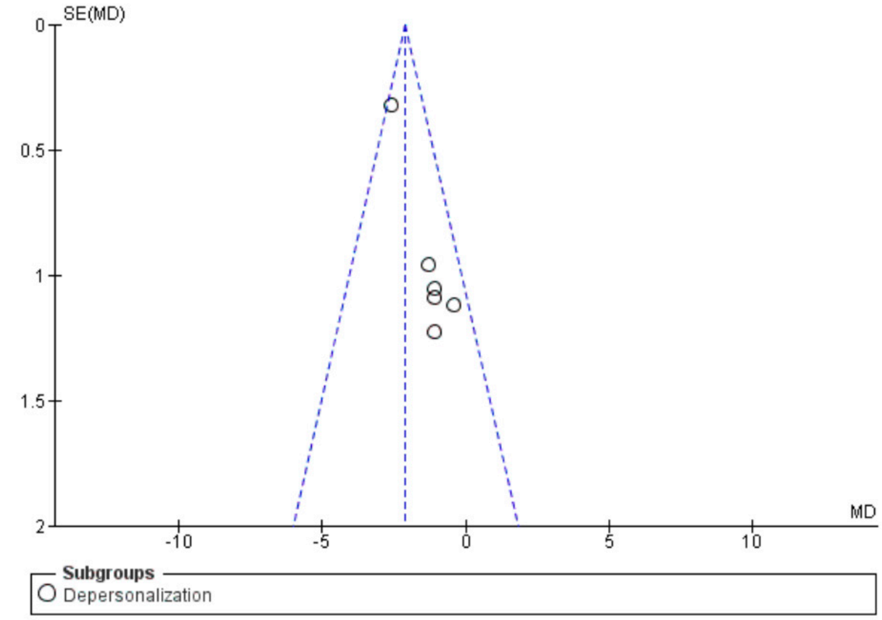

C. Personal Accomplishment (PA).

C.

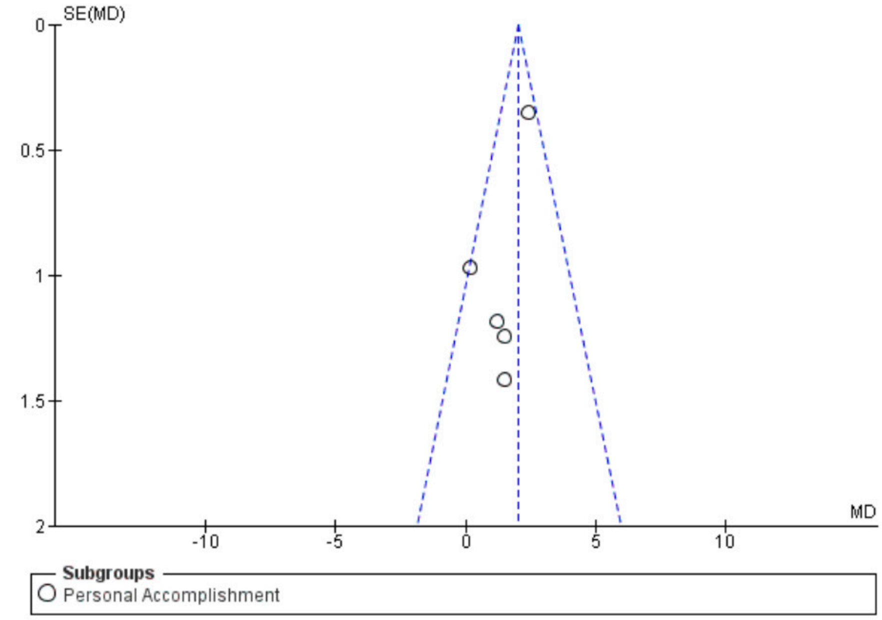

Figure S3. Risk of bias (RoB) summary for intervention studies.

Risk of bias by study across seven domains from the original Cochrane Risk of Bias tool (RoB 1): random sequence generation, allocation concealment, blinding of participants/personnel, blinding of outcome assessment, incomplete outcome data, selective reporting, and other bias. Color indicates rating: Green low risk, White: unclear risk, Red: high risk.

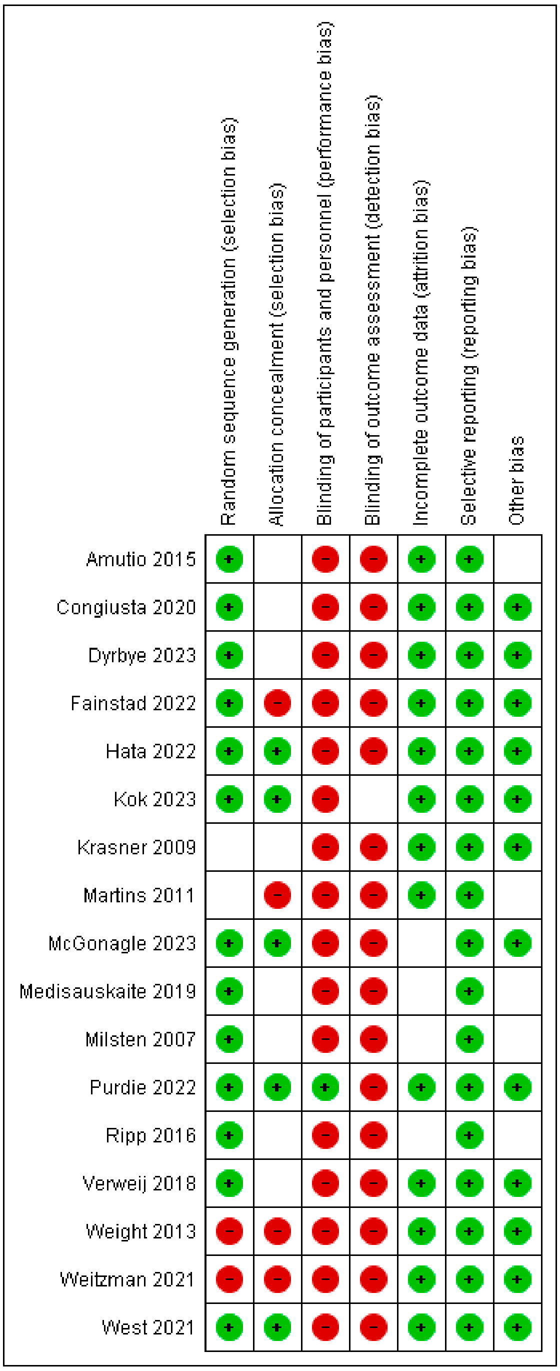

Table S1. Risk of bias judgments for intervention studies.

Risk of bias ratings were assigned for each domain using RoB 1 criteria.

Study	Random sequence	Allocation conceal.	Blinding (partic./ personnel)	Blinding (outcome)	Incomplete data	Selective reporting	Other bias
Krasner 2009	Unclear	Unclear	High	High	Low	Low	Low
Milstein 2009	Low	Unclear	High	High	Unclear	Low	Unclear
Martins 2011	Unclear	High	High	High	Low	Low	Unclear
Weight 2013	High	High	High	High	Low	Low	Low
Amutio 2015	Low	Unclear	High	High	Low	Low	Unclear
Ripp 2016	Low	Unclear	High	High	Unclear	Low	Unclear
Verweij 2018	Low	Unclear	High	High	Low	Low	Low
Medisauskaite 2019	Low	Unclear	High	High	Unclear	Low	Unclear
Congiusta 2020	Low	Unclear	High	High	Low	Low	Low
McGonagle 2020	Low	Low	Unclear	Unclear	Unclear	Low	Low
Weitzman 2021	High	High	High	High	Low	Low	Low
West 2021	Low	Low	High	High	Low	Low	Low
Fainstad 2022	Low	High	High	High	Low	Low	Low
Hata 2022	Low	Low	High	High	Low	Low	Low
Kok 2023	Low	Low	High	Unclear	Low	Low	Low
Dyrbye 2023	Low	Unclear	High	High	Low	Low	Low

Table S2. Characteristics and interventions of studies excluded from meta-analysis due to methodological limitations.

This table summarizes intervention studies screened for quantitative synthesis that were not pooled because MBI subscale values (emotional exhaustion [EE], depersonalization [DP], personal accomplishment [PA]) were not reported in extractable group-level form, were reported on a non-comparable scale, or were reported only as prevalence, change scores, or other non-extractable formats.

Study	Design and population	Intervention and comparator	Burnout measure and reporting	Reason for exclusion
Milstein 2009	Randomized trial; pediatric house officers (single US program); N=15 (7 intervention, 8 control); follow-up 3 months.	Intervention: 45-min training in the BATHE communication tool, then self-use during the study period.<br>Control: no BATHE training; dismissed after baseline survey.	Maslach Burnout Survey administered at baseline and 3 months; results shown graphically without numeric domain values.	Domain scores not extractable (figure-only reporting).
Martins 2011	Randomized trial; pediatric residents at a tertiary pediatric hospital (Buenos Aires, Argentina); N=74 (37 per group); follow-up after 2 months.	Intervention: two 2.5-h workshops led by mental health staff (burnout effects, risk signs, coping and self-care skills).<br>Control: no workshop.	MBI (Spanish validation) used; outcomes reported as burnout prevalence and regression results.	No extractable group-level domain means with dispersion (EE, DP, PA).

Weight 2013	Prospective cohort; physician trainees in a team-based, incentivized exercise program; baseline survey N=628.	Intervention: 12-week team exercise contest with incentives.<br>Comparator: nonparticipants.	Burnout measured with two single-item questions for emotional exhaustion and depersonalization; no personal accomplishment subscale.	Non-standard burnout metric (single-item measures; incomplete MBI domains).
Amutio 2015	Randomized trial; practicing physicians; N=42 (21 per group); 8-week course with later maintenance sessions.	Intervention: Mindfulness-Based Stress Reduction (8 weekly 2.5-h sessions plus one 8-h retreat), then monthly maintenance.<br>Control: no intervention during the main phase.	MBI for health professionals; burnout and subscales reported on a 0-6 item scale as mean scores (with SD).	Non-standard scoring scale relative to pooled analyses using standard subscale totals.
Ripp 2016	Randomized trial; first-year internal medicine residents; N=51 eligible; analysis based on participants completing both surveys.	Intervention: twice-monthly, theme-based facilitated discussion groups (18 one-hour sessions).<br>Control: usual training without groups.	MBI used; burnout reported as high emotional exhaustion or depersonalization (dichotomized).	Outcomes reported as prevalence/incidence only; no domain means with dispersion.
Verweij 2018	Randomized trial; residents at an academic medical center (Netherlands); N=148 (80 intervention, 68 control); postintervention at about 3 months.	Intervention: Mindfulness-Based Stress Reduction (8 weekly 2.5-h sessions plus one 6-h silent day).<br>Control: waitlist/usual practice.	Dutch adaptation of the MBI (UBOS-C) used; item set differs from standard instrument (item deletion).	Instrument variant with different items and scoring from standard MBI.
Medisauskaite 2019	Randomized trial; UK doctors recruited via professional channels; analysis compares group 4 (n=39) vs control (n=52) over 7 days.	Intervention: online modules on psychology of distress and coping (all modules completed within 7 days).<br>Control: no modules.	MBI domains reported on a 0-6 item scale as mean scores (with SD) and as prevalence thresholds.	Domain scores reported on a non-standard scale for pooling with studies reporting standard subscale totals.
McGonagle 2020	Randomized trial with waitlist; primary care physicians; N=59 enrolled; coaching over 3 months.	Intervention: six coaching sessions (first 60 min, then five 30-min sessions, about every 2 weeks; first in person, then phone).<br>Control: waitlist.	MBI administered; reporting focuses on an overall burnout score without separate EE, DP, and PA values.	MBI subscales not reported as extractable domain values.
Weitzman 2021	Prospective randomized crossover trial; otolaryngology residents; N=18; two 8-week phases.	Intervention: 10-min weekly VR-guided meditation with paced breathing.<br>Control: no intervention during the alternate phase.	MBI used; results reported as within-participant change with nonparametric comparisons.	Change-score reporting without extractable group-level domain values at each timepoint.
Hata 2022	Randomized trial; interprofessional clinical faculty (physicians, nurse practitioners,	Intervention: three monthly self-facilitated dinners; randomization to no written guide vs one-page guide.<br>Comparator: alternate arm.	Two single-item measures for emotional exhaustion and depersonalization	Non-standard burnout metric and mixed professional sample.

	certified nurse midwives); N=25.		derived from the MBI; no full domain scores.	
Kok 2023	Parallel cluster randomized trial; six ICUs in two hospitals; N=435 ICU professionals.	Intervention: structural moral case deliberation sessions.<br>Control: no structural moral case deliberation.	MBI on a 0-6 scale; primary outcomes are domain scores for EE, DP, and low personal accomplishment.	Mixed professional ICU sample and non-standard scale relative to pooled analyses.

Abbreviations: EE, emotional exhaustion; DP, depersonalization; PA, personal accomplishment; MBI, Maslach Burnout Inventory; MBI-HSS, Maslach Burnout Inventory–Human Services Survey; MBSR, Mindfulness-Based Stress Reduction; PCP, primary care physician; RCT, randomized controlled trial; VR, virtual reality; SE, standard error; MD, mean difference; RoB, risk of bias.
